# Smoking-related general and cause-specific mortality in Estonia

**DOI:** 10.1186/s12889-017-4590-3

**Published:** 2017-07-19

**Authors:** Gea Kõks, Krista Fischer, Sulev Kõks

**Affiliations:** 1 Department of Pathophysiology, University of Tartu, 19 Ravila Street, 50411 Tartu, Estonia; 2 Estonian Genome Center, University of Tartu, 23b Riia Street, 51010 Tartu, Estonia; 3 Department of Reproductive Biology, Estonian University of Life Sciences, 62 Kreutzwaldi Street, 51006 Tartu, Estonia

**Keywords:** Smoking, Survival, Cause of death, Cancer, Mortality

## Abstract

**Background:**

Tobacco smoking is known to be the single largest cause of premature death worldwide. The aim of present study was to analyse the effect of smoking on general and cause-specific mortality in the Estonian population.

**Methods:**

The data from 51,756 adults in the Estonian Genome Center of the University of Tartu was used. Information on dates and causes of death was retrieved from the National Causes of Death Registry. Smoking status, general survival, general mortality and cause-specific mortality were analysed using Kaplan-Meier estimator and Cox proportional hazards models.

**Results:**

The study found that smoking reduces median survival in men by 11.4 years and in women by 5.8 years. Tobacco smoking produces a very specific pattern in the cause of deaths, significantly increasing the risks for different cancers and cardiovascular diseases as causes of death for men and women. This study also identified that external causes, such as alcohol intoxication and intentional self-harm, are more prevalent causes of death among smokers than non-smokers. Additionally, smoking cessation was found to reverse the increased risks for premature mortality.

**Conclusions:**

Tobacco smoking remains the major cause for losses of life inducing cancers and cardiovascular diseases. In addition to the common diseases, external causes also reduce substantially the years of life. External causes of death indicate that smoking has a long-term influence on the behaviour of smokers, provoking self-destructive behaviour. Our study supports the idea, that tobacco smoking generates complex harm to our health increasing mortality from both somatic and mental disorders.

## Background

Tobacco smoking is a single major cause of premature death worldwide [1, 2]. Despite substantial reduction in the use of tobacco, smoking still causes more deaths globally than diseases like tuberculosis, HIV and malaria combined, making it the largest preventable health risk factor [3, 4]. The use of tobacco is legally allowed and therefore the prevalence of smoking behaviour is still very high. While in the developed countries tobacco use is reduced and restricted, in developing countries the tobacco epidemic is still in the growing phase [1, 5]. It has been estimated that tobacco smoking causes globally 6 million deaths in a year [6]. 80% of these deaths are premature and affect lower income individuals [5]. This all makes tobacco smoking the largest single avoidable cause for mortality. Reducing the prevalence of smoking increases the health of the general population by avoiding premature disability and death. Smoking cessation and support to stop smoking are the easiest tools to improve the quality of life in population [7, 8].

While it is clear that smoking increases mortality, the association between smoking and mortality is different across specific causes of death. Cancers, chronic obstructive diseases of respiratory system and cardiovascular diseases are the most commonly referred smoking-induced causes of death.

The association between smoking and lung cancer has been described already in 1950 and following years [9–12]. Later studies found clear association with other cancers as well [13]. Tobacco smoking is known to cause cancers of upper gastrointestinal tract, pancreas, prostate [14]. While the association is conclusive for lung and upper respiratory tract cancers, the connection between smoking and other cancers does not have conclusive evidence.

Tobacco smoking is reported to cause 90% of all chronic obstructive pulmonary disease (COPD) mortality and smoking cessation is the best measure to avoid this outcome [15]. The relative risk for smokers to die from COPD is 25.6 in men and 22.3 in women, compared to non-smokers [16]. Therefore, one of the most common health problems in smokers is COPD [16, 17]. The development of COPD is dependent on the chronic persistent inflammation in the lung tissue that induces tissue-remodelling [18]. COPD is the most common and the most prevalent disease in smokers and smokers have very highly increased risk for COPD.

Cardiovascular diseases have been found to be the third major cause of death caused by smoking [16, 19]. The main causes for death are ischaemic heart disease, cerebrovascular disease and other heart diseases [16]. Interestingly the risks caused by smoking of death due coronary heart disease are remarkably different between men and women [20]. Women who smoke have four times increased risk of death from ischaemic heart disease, while male smokers only have twofold increased risk [20]. For cerebrovascular diseases, this sex difference in risk has not been found [21]. This influence of sex on the risk of ischaemic heart disease is striking and its mechanism could be biological or behavioural.

Increased prevalence of chronic diseases suggests increased inflammatory activity in smokers. Indeed, several studies indicate chronic inflammation in the smokers and different immunomodulating toxins in the smoke probably cause increased immune reactivity [22, 23]. A recent study analysed RNA expression profiles in the blood of smokers and non-smokers and identified a highly significant increase in the expression of GPR15 gene [24]. GPR15 is an orphan receptor that is responsible for the homing of tissue-specific immune cells [25].

Several previous studies have shown that the association between smoking with different causes of deaths is variable [26]. There are some large-scale population studies where causes of death and association with smoking have been addressed [7, 13, 16]. However, more detailed differential risk analyses for smoking and causes of death are not that common [27]. The goal of present study was to analyse the smoking-caused mortality in the Estonian Genome Center cohort and to describe the differential sensitivity for the different specific causes of death.

## Methods

### The aim, design and setting of the study

The aim of the study was to analyse smoking-induced mortality profile in Estonia using data at the Estonian Genome Center. Estonian Genome Center at the University of Tartu is a population-based biobank that recruited a cohort of 51,756 participants, including adults from all counties in Estonia, accounting for approximately 5% of the Estonian adult population during the recruitment period. Recruitment was performed during the period from 2002 to 2012 [28]. At baseline, an extensive phenotype questionnaire was conducted together with a measurement panel. Follow-up data is available from linkage with national health-related registries and the Estonian Health Insurance database. Mortality information was retrieved from the Estonian Causes of Death Registry and last retrieval was performed in September 2015.

The Ethics Review Committee on Human Research of the University of Tartu approved the protocols and informed-consent forms used in this study. All of the participants signed a written informed-consent form.

### Smoking data

Smoking data were collected during the recruitment of donors to the Estonian Genome Center. The following questions were asked:

1. “If you have ever smoked regularly, when did you start?”
2. “How many of the tobacco products have you used during the last 12 months?”
3. “For how many years have you used tobacco products with this amount?”
4. “What is the amount of tobacco products you used most commonly?”
5. “For how many years you have used tobacco products as described?”

Smoking behaviour was divided into three major categories: current smokers, former smokers and never smoked. Former smokers were individuals who had not smoked for at least one year prior to recruitment.

### Statistical analysis

All statistical analyses were stratified by sex, as previous analysis indicated significant differences in the smoking patterns between males and females. Statistical analysis was performed in R studio and packages “survival”, “epitools”, “Publish”, “dplyr” were used. Survival data were modelled by using Cox model [29]. Survival was calculated from the date when subjects joined the Estonian Genome Centre cohort (2002–2012) and censored at date of death, retrieved from the National Death Registry, or September 2015, whichever came first.

The data on smoking status, smoking intensity and smoking pattern was categorized (current, former and never smokers; smoking up to 10, 10–20 or more than 20 cigarettes per day; up to 20 years or more than 20 years of smoking history) and tabulated by four age groups (18–25, 26–45, 46–65 and more than 65 years).

Mortality data was tabulated according to the cause of death, smoking status and sex of all participants. For survival analysis, the Kaplan-Meier estimator was used to plot survival curves. Starting time-point was the date when subjects joined the Estonian Genome Centre cohort and the end-point was the date of data retrieval from the National Death Registry. Cox proportional hazards regression analysis was used to calculate hazard ratio for all-cause mortality and cause-specific mortality, comparing current and former smokers with never-smokers, using age as a time scale (to account for left-truncation in the data). Cox regression was also used to estimate the effects of smoking intensity (cigarettes per day) and length of smoking history on overall survival. Using the Kaplan-Meier method for left-truncated data, the median survival time for current, former and never smokers in the cohort also was estimated with a 95% confidence interval.

For cause-specific mortality, the causes of death according to the 10th Revision of International Statistical Classification of Diseases and Related Health Problems (ICD-10) were analysed separately in men and women.

## Results

### General smoking characteristics in the cohort

The Estonian Genome Center includes 51,756 participants: 17,777 men and 33,979 women (Table 1), aged 18 and over at recruitment. The recruitment period was between 2002 and 2012.

**Table 1:** Age distribution in the cohort of the Estonian Genome Center of the University of Tartu 2002–2012

	Men		Women		Total	
Variable	n	%	n	%	n	%
Age group						
18–25	3896	7.5	5302	10.3	9198	17.8
26–45	6016	11.6	12,436	24.0	18,452	35.6
46–65	5292	10.2	11,257	21.8	16,549	32.0
over 65	2573	5.0	4984	9.6	7557	14.6
Total	17,777	34.3	33,979	66.7	51,756	100.0

Basic demographic information and smoking characteristics are given in the Tables 2 and 3. Of all 17,777 men (Table 2), 39.4% were never-smokers, 20.8% former and 39.8% current smokers. Of all 33,979 women (Table 3), 67.3% were never-smokers, 10.0% former and 22.7% current smokers. This indicates clearly higher prevalence of smoking in Estonian men than women. The proportion of smokers in the cohort is similar to that of the general Estonian population [1]. According to the recent WHO data, the smoking prevalence in Estonian men is 41.2% and in women 24.9% [1].

**Table 2:** Smoking characteristics by age-group in the male cohort of the Estonian Genome Center of the University of Tartu 2002–2012

	Age group									
	18–25		26–45		46–65		Over 65		Total	
Variable	n	%	n	%	n	%	n	%	n	%
Smoking status										
Total	3896	100.0	6016	100.0	5292	100.0	2573	100.0	17,777	100.0
Never smoker	1854	47.6	2281	37.9	1783	33.7	1093	42.5	7011	39.4
Former smoker	218	5.6	1013	16.8	1409	26.6	1058	41.1	3698	20.8
Current smoker	1824	46.8	2722	45.3	2100	39.7	422	16.4	7068	39.8
Age at start (SD)	16	(2.4)	18	(3.5)	19	(4.5)	20	(5.4)	18	(4.2)
	Current smokers									
Years smoked (SD)	5	(2.8)	17	(6.5)	35	(7.1)	51	(8.0)	108	(14.7)
Up to 20 years	1773	100.0	1772	66.8	52	2.5	4	0.9	3601	52.3
Over 20 years	0	0.0	879	33.2	1991	97.5	409	99.1	3279	47.7
Cigarettes per day										
Number of persons	1807	100.0	2698	100.0	2084	100.0	419	100.0	7008	100.0
Up to 10	634	35.1	551	20.4	299	14.4	102	24.3	1586	22.6
10–20	1101	60.9	1898	70.4	1555	74.6	295	70.4	4849	69.2
Over 20	72	4.0	249	9.2	230	11.0	22	5.3	572	8.2
	Former smokers									
Years smoked (SD)	4	(2.6)	11	(6.5)	22	(11.3)	29	(15.2)	20	(13.7)
Up to 20 years	192	100.0	893	91.2	645	47.1	340	33.0	2070	58.0
Over 20 years	0	0.0	86	8.8	724	52.9	691	67.0	1501	42.0
Cigarettes per day										
Number of persons	163	100.0	766	100.0	1095	100.0	869	100.0	2893	100.0
Up to 10	87	53.4	184	24.0	161	14.7	126	14.5	558	19.3
10–20	72	44.2	515	67.2	791	72.3	661	76.1	2039	70.5
Over 20	4	2.4	67	8.8	143	13.0	82	9.4	296	10.2

**Table 3:** Smoking characteristics by age-group in the female cohort of the Estonian Genome Center of the University of Tartu 2002–2012

	Age group									
	18–25		26–45		46–65		Over 65		Total	
Variable	n	%	n	%	n	%	n	%	n	%
Smoking status										
Total	5302	100.0	12,436	100.0	11,257	100.0	4984	100.0	33,979	100.0
Never smoker	3390	63.9	7587	61.0	7471	66.4	4407	88.4	22,855	67.3
Former smoker	328	6.2	1374	11.0	1334	11.8	366	7.3	3402	10.0
Current smoker	1584	29.9	3475	28.0	2452	21.8	211	4.3	7722	22.7
Age at start (SD)	16	(2.2)	20	(4.4)	23	(7.3)	27	(10.0)	21	(6.3)
	Current smokers									
Years smoked (SD)	5	(2.7)	16	(6.2)	29	(8.3)	42	(11.0)	92	(11.7)
Up to 20 years	1540	100.0	2556	76.4	314	13.1	10	4.8	4420	
Over 20 years	0	0.0	790	23.6	2076	86.9	197	95.2	3063	
Cigarettes per day										
Number of persons	1570	100.0	3435	100.0	2436	100.0	211	100.0	7652	100.0
Up to 10	968	61.7	1510	44.0	1020	41.9	104	49.3	3602	47.0
10–20	584	37.2	1842	53.6	1363	55.9	104	49.3	3893	51.0
Over 20	18	1.1	83	2.4	53	2.2	3	1.4	157	2.0
	Former smokers									
Years smoked (SD)	3	(2.4)	9	(6.1)	18	(10.7)	24	(14.9)	14	(10.9)
Up to 20 years	293	100.0	1210	94.7	768	60.9	162	45.6	2433	76.3
Over 20 years	0	0.0	68	5.3	494	39.1	193	54.4	755	23.7
Cigarettes per day										
Number of persons	259	100.0	1051	100.0	1049	100.0	300	100.0	2659	100.0
Up to 10	183	70.6	573	54.5	571	54.4	160	53.3	1487	55.9
10–20	75	29.0	470	44.7	458	43.7	136	45.3	1139	42.9
Over 20	1	0.4	8	0.8	20	1.9	4	1.4	33	1.2

The average age to start smoking in men was 18 (SD 4.2) years and in women 21 (SD 6.3) years. Analysis of the different age groups indicated that nowadays people start smoking at a younger age than in the past. Men in the age group 65+ had started smoking at age 20 and women at age 27 in the average. On the other hand, average age of starting to smoke in the youngest age group (18–25) was 16 for both, men and women.

The cohort was divided into two groups according to the duration of smoking – up to 20 years and over 20 years of smoking. In the study cohort, most of the smokers were smoking for less than 20 years. This trend also was valid in all subgroups of the cohort.

The number of cigarettes per day differed significantly between male and female smokers. On average, men smoked 15 (SD 9) and women smoked 9 (SD 7) cigarettes per day. The average number of cigarettes per day was similar in current and former smokers.

### All-cause mortality

By September 2015, 3364 (1649 men and 1715 women) people the cohort had died. The average age at death was 69 (SD 14) and 74 (SD 14) years for men and women, respectively. A general description of the more frequent causes of death and their association with smoking is given in the Table 4.

**Table 4:** Basic characteristics of smoking, number of all causes of death and the most common cause-specific deaths in the studied cohort

	Variable	Men			Women		
	Smoking status	Current	Former	Never	Current	Former	Never
		n	n	n	n	n	n
	Persons in cohort	7068	3698	7011	7722	3402	22,855
	All causes death in cohort	627	548	474	270	147	1298
C16	Malignant neoplasm of stomach	8	13	10	10	5	34
C18	Malignant neoplasm of colon	3	10	9	4	1	25
C25	Malignant neoplasm of pancreas	11	18	11	12	6	32
C34	Malignant neoplasm of bronchus and lung	73	42	5	15	6	19
C50	Malignant neoplasm of breast	–	–	–	12	8	35
C56	Malignant neoplasm of ovary	–	–	–	5	8	25
C61	Malignant neoplasm of prostate	16	17	29	–	–	–
C80	Malignant neoplasm without specification of site	9	–	2	4	–	9
I11	Hypertensive heart disease	41	41	39	20	22	190
I13	Hypertensive heart and renal disease	8	10	13	5	4	28
I21	Acute myocardial infarction	16	11	12	6	4	31
I22	Subsequent myocardial infarction	12	9	2	2	2	11
I25	Chronic ischaemic heart disease	80	113	100	21	21	243
I35	Nonrheumatic aortic valve disorders	5	2	6	1	1	17
I42	Cardiomyopathy	16	9	12	5	2	11
I50	Heart failure	10	2	1	4	1	17
I63	Cerebral infarction	16	30	13	9	9	79
I70	Atherosclerosis	2	3	5	2	1	11
J15	Bacterial pneumonia	1	4	3	3	2	7
J44	Chronic obstructive pulmonary disease	17	18	2	7	1	10
K70	Alcoholic liver disease	8	7	6	7	0	2
R99	Ill-defined and unspecified causes of mortality	6	3	4	5	0	2
X45	Accidental poisoning by and exposure to alcohol	14	1	3	2	0	1
X70	Intentional self-harm by hanging, strangulation and suffocation	13	3	8	5	1	5

**Fig. 1:**
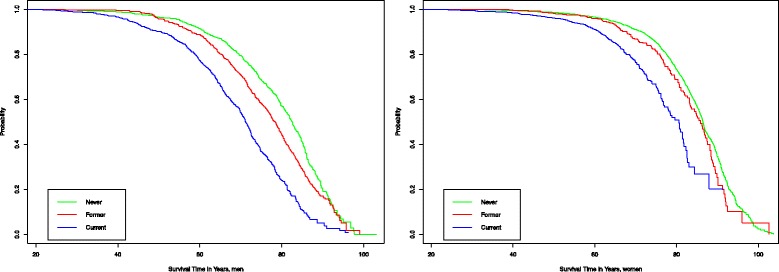
Kaplan-Meier survival probabilities for current and former smokers and for men and women who have never smoked. Reduced survival of the smokers is evident

The Kaplan-Meier curves for overall survival were significantly different in current, former and never smokers, in both men and women (Fig. 1). The test for survival curve differences in men between three different smoking categories (“Never”, “Former”, “Current”) was highly significant (*p* = 1.8*10^−40^). In case of women, the difference was also highly significant (*p* = 1.3*10^−14^) for three-group comparison. The never smoked group had the longest survival and current smokers had the shortest survival. Survival of former smokers was in-between the never and current smokers indicating beneficial effect of quitting.

Age-adjusted Cox regression of all-cause mortality in both sexes indicated significantly increased hazard ratio (HR) and 95% confidence interval (CI), for current 3.1 (CI 2.8–3.4) and former 1.6 (CI 1.5–1.8) smokers, compared to never smokers. When different sexes were analysed separately, almost similar results were seen. In male current smokers HR was 2.5 (CI 2.2–2.8) and in former smokers 1.3 (CI 1.2–1.5) for all-cause mortality. Currently smoking women had HR 2.6 (CI 2.2–3.0) and former smokers had 1.3 (CI 1.1–1.5) for all-cause mortality. Taken together, smoking status was significantly associated with increased all-cause mortality and this association was similar for men and women.

### Median age at death and loss of life years

Smoking significantly reduced median age of death. In the entire study group, median age of death in never smokers was 85.7 (CI 85.4–86.2), in former smokers 80.6 (CI 79.6–81.6) and in current smokers 74.7 (CI 73.5–75.7) years.

The median age of death for men who never smoked was 82.6 (CI 81.4–83.6), for former smokers 78.4 (CI 77.1–79.4) and for current smokers 71.2 (CI 70.2–72.3) years. Median age of death for women who never smoked was 86.4 (CI 86.1–86.8), for former smokers 85.8 (CI 83.4–88.1) and for current smokers 80.6 (CI 77.9–81.8) years.

Therefore, we estimate that the median loss of life years in smoking men is 11.4 years and in women 5.8 years at lost. In the case of men, former smokers lived significantly longer than current smokers, but significantly less than never smokers. In the case of women, the difference in age of death of former and never smokers were very small.

### Effect of smoking duration and smoking intensity on all-cause mortality

The increased risk for all-cause mortality was also significantly associated with smoking duration. Men who smoked more than 20 years had HR 2.1 (CI 1.9–2.3) and men who smoked less than 20 years had HR 2.3 (CI 2.1–2.5). Women with smoking duration more than 20 years had HR 2.7 (CI 2.4–3.1) and with less than 20 years had HR 1.4 (CI 1.3–1.6). However, the number of cigarettes per day had a less severe influence on the all-cause mortality than smoking duration.

### Cause-specific mortality

The subsequent analysis involved specific causes for mortality. The study cohort altogether had 272 unique ICD-10 main categories as cause of death. The most frequent causes of death (more than 100 cases) were chronic ischaemic heart disease (I25), hypertensive heart disease (I11), malignant neoplasm of bronchus and lung (C34) and cerebral infarction (I63) across the entire cohort (Table 4). Men and women also were analysed separately and for specific causes of death age-adjusted hazard ratio was calculated with non-smokers as reference. Statistically significant findings are shown in Tables 5 and 6.

**Table 5:** Smoking related adjusted hazard ratio (HR) and 95% confidence intervals (CI 95%) for the statistically significant cause-specific mortality in men

	Variable	Current smokers		Former smokers	
	ICD-10 code for cause of death	HR	CI 95%	HR	CI 95%
C34	Malignant neoplasm of bronchus and lung	32.9	13.2–82.0	9.2	3.7–23.4
C80	Malignant neoplasm without specification of site	7.8	1.5–39.5	–	–
C16–C80	Malignant neoplasms	4.0	2.1–5.4	1.7	1.2–2.3
I11	Hypertensive heart disease	2.1	1.3–3.3	1.2	0.8–1.8
I22	Subsequent myocardial infarction	9.4	2.0–43.3	5.4	1.2–24.8
I25	Chronic ischaemic heart disease	1.9	1.4–2.6	1.3	0.9–1.7
I50	Heart failure	24.4	2.8–213.1	2.1	0.2–23.4
I63	Cerebral infarction	3.3	1.5–7.0	2.7	1.4–5.3
I00–I99	Diseases of the circulatory system	2.6	1.8–2.7	1.3	1.1–1.5
J44	Chronic obstructive pulmonary disease	23.1	5.2–101.8	9.7	2.3–41.7
J00–J99	Diseases of the respiratory system	7.8	3.2–18.7	4.2	1.8–9.7
X45	Accidental poisoning by and exposure to alcohol	3.6	1.1–12.7	0.5	0.05–5.1
V01–Y98	External causes of morbidity and mortality	2.2	1.5–3.3	0.8	0.5–1.4

**Table 6:** Smoking related adjusted hazard ratio (HR) and 95% confidence intervals (CI 95%) for the statistically significant cause-specific mortality in women

	Variable	Current smokers		Former smokers	
	ICD-10 code for cause of death	HR	CI 95%	HR	CI 95%
C25	Malignant neoplasm of pancreas	4.0	1.9–8.3	1.9	0.8–4.7
C34	Malignant neoplasm of bronchus and lung	6.7	3.2–14.4	3.0	1.2–7.6
C56	Malignant neoplasm of ovary	0.9	0.3–2.5	2.4	1.1–5.3
C16–C80	Malignant neoplasms	2.7	1.9–3.7	1.8	1.2–2.6
I11	Hypertensive heart disease	2.2	1.3–3.6	1.4	0.9–2.2
I13	Hypertensive heart and renal disease	3.3	1.1–9.8	1.7	0.6–4.9
I21	Acute myocardial infarction	4.7	1.9–12.2	1.6	0.6–4.6
I25	Chronic ischaemic heart disease	2.1	1.3–3.3	1.1	0.7–1.7
I50	Heart failure	10.3	2.9–35.8	0.9	0.1–6.6
I63	Cerebral infarction	3.4	1.6–7.0	1.6	0.8–3.1
I70	Atherosclerosis	10.2	1.8–57.4	1.2	0.2–9.6
I00–I99	Diseases of the circulatory system	2.7	2.1–3.5	1.3	1.0–1.7
J15	Bacterial pneumonia	6.4	1.3–32.2	3.2	0.7–15.8
J44	Chronic obstructive pulmonary disease	19.3	7.1–52.6	1.3	0.2–10.2
J00–J99	Diseases of the respiratory system	12.0	5.3–27.3	1.8	0.5–6.1
K70	Alcoholic liver disease	13.9	2.6–75.8	–	–
R99	Ill-defined and unspecified causes of mortality	8.5	1.6–45.6	–	–
X70	Intentional self-harm by hanging, strangulation and suffocation	4.6	1.2–18.1	1.6	0.2–13.7
V01–Y98	External causes of morbidity and mortality	7.6	3.9–14.7	2.6	1.0–6.5

**Fig. 2:**
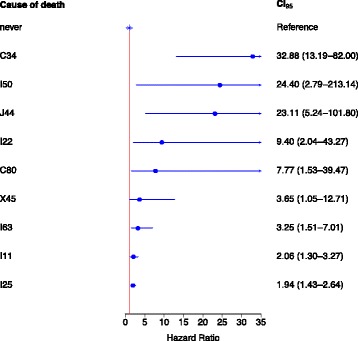
Forest plot of the hazard ratio (HR) for specific causes of death illustrates the profile of the major causes of death in currently smoking men. Age-adjusted Cox regression modelling was applied

The most significant specific causes for mortality in currently smoking men were malignant neoplasm of bronchus and lung (C34, HR 32.9), heart failure (I50, HR 24.4), chronic obstructive pulmonary disease (J44, HR 23.1) and subsequent myocardial infarction (I22, HR 9.4) (Table 5, Fig. 2). Current smokers also had a significantly increased hazard ratio to die from malignant neoplasms at unspecified sites (C80, HR 7.8). Interestingly, current smokers also had a significantly elevated hazard ratio for accidental poisoning by an exposure to alcohol (X45, HR 3.6), for cerebral infarction (I63, HR 3.3), for hypertensive heart disease (I11, HR 2.1) and chronic ischaemic disease (I25, HR 1.9) as causes of death.

Compared to current smokers, former male smokers (Table 5) had significantly lower hazard ratio values for most of the causes of death. This finding illustrates a clear health benefit of quitting smoking as the general risk for death reduced from 2.5 to 1.3 and the number of specific causes for death decreased. In men who stopped smoking, only four causes of death remained statistically significant compared to those who have never smoked. These causes of death were chronic obstructive pulmonary disease (J44, HR 9.7), malignant neoplasm of bronchus and lung (C34, HR 9.2), subsequent myocardial infraction (I22, HR 5.4) and cerebral infarction (I63, HR 2.7).

**Fig. 3:**
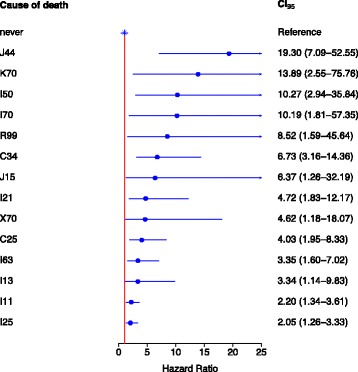
Forest plot of the hazard ratio (HR) for specific causes of death illustrates the profile of the major causes of death in currently smoking women. Age-adjusted Cox regression modelling was applied

In the case of currently smoking women (Table 6, Fig. 3) the highest hazard ratio as a cause of death were for chronic obstructive pulmonary disease (J44, HR 19.3), for alcoholic liver disease (K70, HR 13.9), for heart failure (I50, HR 10.3) and for atherosclerosis (I70, HR 10.2). Current smokers also had increased hazard ratio for ill-defined and unspecified causes of mortality (R99, HR 8.5), for malignant neoplasm of bronchus and lung (C34, HR 6.7), for bacterial pneumonia (J15, HR 6.4), for acute myocardial infarction (I21, HR 4.7) and intentional self-harm by hanging, strangulation and suffocation (X70, HR 4.6) as a cause of death. In currently smoking women, the hazard ratio for accidental poisoning by an exposure to alcohol (X45) was elevated, but this increase did not reach statistical significance.

Smoking cessation significantly reduced the risks for specific causes of death and most of the hazard ratios were not different compared to the never smoked group (Table 6). In former smokers only malignant neoplasm of the bronchus and lung (C34, HR 3.0) and malignant neoplasm of the ovaries (C56, HR 2.4) remained significant specific causes of death.

Taken together, current smokers have significantly increased mortality and a specific pattern of cause of death. Respiratory and cardiovascular diseases were the most significant causes of death. This indicates, that smoking does the most harm to the lungs and cardiovascular system. The former smokers group had significantly less mortality than current smokers and therefore, smoking cessation significantly reduces the hazard ratio for cause-specific mortality. Former smokers still have significantly increased hazard to die from the lung diseases.

## Discussion

Based on the epidemiological analysis of tobacco smoking in the cohort of the Estonian Genome Center, this study concluded that Estonia still belongs to the group of countries with high number of smokers. On average, there are two times more smokers in Estonia than in Western-European countries. The high number of smokers also is characteristic for other Eastern-European countries. This statement is particularly true in men. In Estonia and other Eastern-European countries around 40% of men are smokers, whereas in Western-European countries only 10–20% of men are smokers. In the case of female smokers, Estonia is similar to Western-European countries, where 20% of women smoke [1]. This fits the typical epidemiologic pattern seen in Europe and developed countries. Differences between different countries are smaller in case of women and are prominent in the case of men. These differences also explain the significantly worse health of men in Eastern-European countries. Men smoke too much and they pay for that with their health and quality of life as seen by the reduced life span.

The smoking prevalence in the Estonian Genome Center cohort is similar to the prevalence described in other study based on general population in three Baltic states [30]. In addition, the age-group prevalence of the Estonian Genome Center cohort is similar to the general Estonian population [28]. Therefore, our results are generalizable for the entire Estonian population.

While the proportion of smokers in Estonia has been stable for 20 years, the age at which individuals start smoking tobacco has decreased remarkably. At present, people start to smoke at the median age of 16 and this is similar for men and women. This trend is worrisome and has an important impact on the health of the Estonian population.

The age-group analysis indicated that men start to quit smoking at the age 46–65, women start quitting 26–45. This indicates that women smoke for a shorter period of their life. Taken together, men start smoking earlier, they smoke more cigarettes per day (mostly 20 cigarettes per day) and they quit smoking later. At the same time, women smoke less (mostly 10 cigarettes per day) they start smoking later and give up smoking earlier. This difference could also be detectable as the difference in survival and causes of death. Therefore smoking has a significantly larger impact on men’s health than in women.

There present study analysed the impact of tobacco smoking on the hazard of all-cause and cause-specific mortality. Survival analysis clearly indicated shorter median survival in smokers compared to non-smokers. Survival analysis also indicated that smoking cessation is beneficial for the survival. This means that toxic effects of smoking are reversible. The positive effect of quitting on the survival has been described in many previous studies [16, 17, 19].

Interestingly, in women the general survival differences caused by tobacco smoking were less prominent. For example, men who smoked lived on average 11.4 years less than non-smokers, bud women who smoked only lost 5.8 years on average. In some previous studies, similar sex-differences in smoking effects have been found [31]. This sex-difference may be explained by the lower smoking activity in women. In our sample women smoked 9 cigarettes per day while men smoked 15 cigarettes per day on average. Women also start smoking later and quit earlier that makes their smoking duration shorter and this explains the reduced number of cigarettes smoked by women. Previous studies support this idea. If women smoke more they have mortality and morbidity profile similar to men and the sex differences disappear [32, 33].

The loss of years found from the survival analysis in the present study was similar to the findings from previous studies where loss of at least 10 years was found [17]. This indicates that the study design used and the results are comparable with other studies. For example, the study of Jha et al. analysed survival from the age 25 [17]. The present study included people from the age 18 and mortality data were retrieved from the National Death Registry. This approach has been used for many previous studies.

The impact of smoking duration on general mortality gave somewhat controversial results as in men we found increased mortality in both groups compared to non-smokers. In case of men, the difference in general mortality between smoking more than 20 years and less than 20 years was very small (HR 2.1 versus 2.3) and statistically not significant. In case of women, the difference related to smoking duration was significant. Women smoking more than 20 years had HR 2.7 (CI 2.4–3.1) and women smoking less than 20 years have HR 1.4 (CI 1.3–1.6). This finding is similar to the previously published findings [34]. Similarly, we found that number of cigarettes per day does not have impact on the all-cause mortality.

The cause-specific analysis of death should describe the most common reasons why smokers lose years of their life. The comparison of causes of death induced by smoking in men and women identified very high similarities between both sexes in the pattern of cause of death. Namely, the most significantly smoking-associated causes of death were diseases of the respiratory and cardiovascular systems both in men and women. This indicates that there is a common biological mechanism behind the smoking that increases morbidity and mortality. This mechanism is the same in men and women. One very good candidate for this common pathway is the GPR15 that was recently found to be upregulated in both male and female smokers [24].

In addition to the lung and heart diseases, the one of the largest hazard ratio was related to external causes of death. In men, this cause was death from accidental poisoning by an exposure to alcohol (X45). In women, these causes were alcoholic liver disease (K70) and intentional self-harm by hanging, strangulation, suffocation (X70). Alcoholic liver disease (K70) is closely related to a poisoning by an exposure to alcohol (X45) and should be taken as an external cause of disease. The significantly increased risks for external causes of death have been described in few previous studies and more detailed analysis is difficult to find [14]. The prevalence of alcohol poisoning and self-harm in former smokers as causes of death were the same in former smoker and non-smokers. This means that the increased incidence of suicide and alcohol intoxication in current smokers could reflect particular biologically determined behavioural endophentoype and personality characteristics of current smokers. Smokers could be more impulsive or more prone to risk taking. One side of this behaviour is increased alcohol consumption, binge drinking and suicide. Not many studies indicate causes for this kind of association, but most probably there is common genetic variation between smokers and suicide victims. One recent study found an association between the polymorphic variable number tandem repeat (VNTR) in the SLC6A4 gene and increased risk for tobacco use disorder [35]. The association between polymorphisms in the BDNF gene and nicotine dependence also has been described [36]. Moreover, as tobacco smoking behaviour is often explained in conjunction with mood disorders, the link between suicide and smoking is not that surprising. At least one deletion in the GSTM1 and GSTT1 genes has been described in anxious smokers while no association with mood disorders have been found [37].

One of the limitations of the study is the small number of several specific causes of death that could make our analysis imprecise. This small number is caused by the short time for observation. Currently it is impossible to get better estimations and this study can be a starting point for the repeated mortality analysis of the Estonian Genome Center cohort. Extended observation will eventually increase the number of specific causes and improve the precision of the analysis. In order to improve the regression analysis, we merged some cases (like J44, J45 and J47) that increased the power to estimate proportional hazard for smokers and ex-smokers. This helped to get more reliable HR estimations.

## Conclusions

In conclusion, the prevalence of tobacco smoking is still high in Estonia and men smoke at least two times more than women. This high prevalence is reflected in substantial loss of years of life and shorter survival of men. Smoking cessation can reverse the increased mortality, but the time needed for this reversal is not known. The two main types of cause of death in smokers were diseases of lung and cardiovascular system. The third group of cause of death is self-destructive behaviour. This finding suggests a common biological substrate in the brain of nicotine addicts and risk-taking behaviour. Better knowledge about the link between psychiatric illnesses and smoking could help to develop more effective interventions. Further studies are required to understand the genetic and biological background of this association.
